# Observation of high ionic (Li^+^ and Na^+^) conductivity in novel hybrid membranes based on 2D lanthanide MOFs

**DOI:** 10.1039/d6ra06654b

**Published:** 2026-09-11

**Authors:** Aparna Potdar, Vaibhav Phopse, Dev Kumar Thapa, Chetana Patil, Soumava Biswas

**Affiliations:** a Department of Research & Development, Dr. Vishwanath Karad MIT World Peace University Survey No, 124, Paud Rd, Kothrud Pune 411038 Maharashtra India; b Department of Chemistry, Dr. Vishwanath Karad MIT World Peace University Survey No, 124, Paud Rd, Kothrud Pune 411038 Maharashtra India biswassoumava@gmail.com soumava.biswas@mitwpu.edu.in; c Chemical Engineering and Process Development Division, CSIR-National Chemical Laboratory Dr. Homi Bhaba Road Pune-411008 India

## Abstract

In this work, for the first time, hybrid membranes are successfully fabricated using 2D lanthanide metal–organic frameworks, PVDF-HFP (poly(vinylidene fluoride-*co*-hexafluoropropylene)), and metal salts (Li^+^ and Na^+^). These hybrid membranes show a layered structure with excellent thermal stability and good mechanical strength. Ionic conductivities in the order of 10^−4^ S cm^−1^ are achieved for both Li^+^ and Na^+^ ions in these membranes. Additionally, the overall ion-conduction behaviour is correlated with the structural characteristics to predict the ion-conduction pathway and mechanism.

## Abstract 2

Novel hybrid membranes made from 2D lanthanide MOFs and PVDF-HFP exhibit excellent dual (Li^+^ and Na^+^) ionic conductivity. This is the first report of lanthanide MOF-based hybrid membranes designed for both Li^+^ and Na^+^ conduction in a single system.

## Graphical abstract

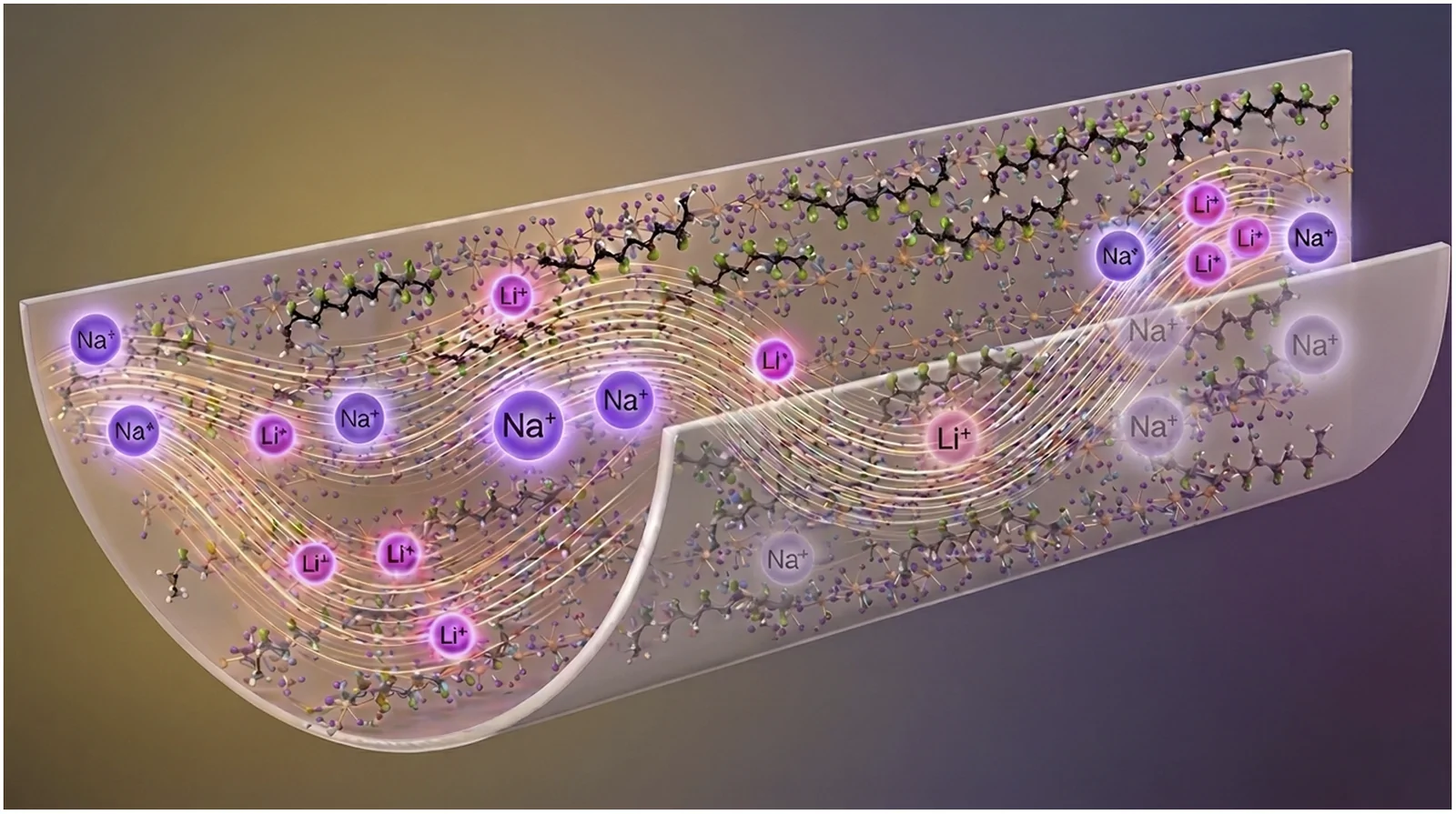

Battery technology must evolve through persistent research efforts to tackle global energy challenges in a greener and more sustainable manner.^1,2^ In this context, it is evident that lithium-ion batteries (LIBs) have been gaining research interest due to their excellent applicability to a wide range of electronic devices, from small to large electric or hybrid vehicles.^3–5^ LIBs have the edge due to the low density, low standard reduction potential, and good specific capacity of lithium metal itself.^6–8^ Apart from that, LIBs show excellent cycling stability, a low self-discharge rate, and a wide operating voltage. However, solid-state electrolytes (SSEs) used in LIBs often suffer from the issues of low ionic conductivity, low thermal stability, and crumbliness and dendrite formation.^9–11^ In this scenario, using MOF-based hybrid membranes (HMs) as SSEs could be a convenient strategy for LIBs to tackle the mentioned issues.^12–14^ The robust reticular structure of MOFs can be firmly implanted in the flexible polymeric matrix of organic polymers (*e.g.*, PVDF-HFP) to produce high-performance SSEs.^15–17^ By the way, some distinct efforts have already been made to fabricate hybrid membranes by combining MOF, PVDF-HFP, and lithium salts. Such MOF-based hybrid membranes show decent ionic conductivity as well.^18–20^

However, it is surprising that no lanthanide MOF (LMOF) has yet been used to create hybrid membranes for metal ion (Li^+^ or Na^+^) conductivity, even though several LMOFs have shown excellent conductivity for proton (H^+^) conduction.^21–24^ Therefore, preparing LMOF-based hybrid membranes for lithium-ion conduction could be a unique strategy.

On the other hand, given the growing demand for lithium in the electric vehicle (EV) industry and the limited global reserves of lithium, finding alternatives is always important. Sodium-based battery technology could be considered as a potential alternative in the present scenario.^25^

Therefore, it is important to develop a clear strategy for creating versatile hybrid membranes for both Li^+^ and Na^+^ ion conduction. Surprisingly, to date, no effort has been made to create such a hybrid membrane for dual-ion conduction. Therefore, LMOF-based hybrid membranes must be investigated for sodium-ion conduction as the lanthanide contraction effect and charge density can create tailored pore channels that selectively facilitate Na^+^ transport. In this work, we report the development of hybrid membranes incorporating two-dimensional isostructural LMOFs, {[Ln_2_(CBDCA)_3_(H_2_O)_2_]_*n*_ 2*n*CH_3_OH·2*n*H_2_O} (Ln = Gd, Tb and Dy, CBDCA = cyclobutane-1,1-dicarboxylate), as functional fillers. Notably, this is the first time LMOFs have been used in this manner. We chose these 2D LMOFs for their excellent proton (H^+^) conduction behaviour^26^ and because the interlayer spacing between 2D LMOFs could be a potential conduction pathway for different ions. It has been observed that several 2D LMOFs have shown excellent proton (H^+^) conduction behaviour. The interlayer spacing can accommodate guest molecules, which can interact with the polymer matrix and coordinated solvents to facilitate ion migration (specifically ion hopping) through the hybrid membrane.^24,26^ This work provides the initial insights into the role of lanthanide MOF structures in constructing PVDF-HFP-based hybrid membranes with conduction of different ions (Li^+^/Na^+^), demonstrating their versatility for advanced solid-state electrolyte applications. Here, all the prepared hybrid membranes show excellent ionic conductivities for both Li^+^ and Na^+^ ions in the order of 10^−4^ S cm^−1^, for the temperature range of 25 °C to 55 °C. Also, these flexible hybrid membranes exhibit good thermal and mechanical stability with heat-resistant properties. Fig. 1 illustrates the preparation of hybrid membranes consisting of the PVDF-HFP polymer (70 wt%), lithium bis(trifluoromethanesulfonyl)imide (LITFSI) or sodium perchlorate (NaClO_4_) (20 wt%), and LMOFs (10 wt%). Acetonitrile was used as the solvent during the synthesis process. Initially, the PVDF-HFP polymer and lithium salt were dissolved in acetonitrile under continuous stirring until a homogeneous solution was obtained. Subsequently, the desired amount of LMOF was incorporated into the solution and uniformly dispersed. The resulting mixture was then cast onto a Petri dish using a solution-casting method. Finally, the membrane was dried in a vacuum oven at 60 °C to remove the solvent and obtain the composite polymer electrolyte film. The digital photograph of the resulting film, as shown in Fig. 1, shows its self-standing and flexible nature, which indicates good compatibility between the polymer matrix and the MOF filler. The lithium-salt-incorporated membranes are denoted as Dy-MOF@LiHM, Gd-MOF@LiHM, and Tb-MOF@LiHM, while the sodium-salt-incorporated membranes are labelled as Dy-MOF@NaHM, Gd-MOF@NaHM, and Tb-MOF@NaHM, respectively.

**Fig. 1:**
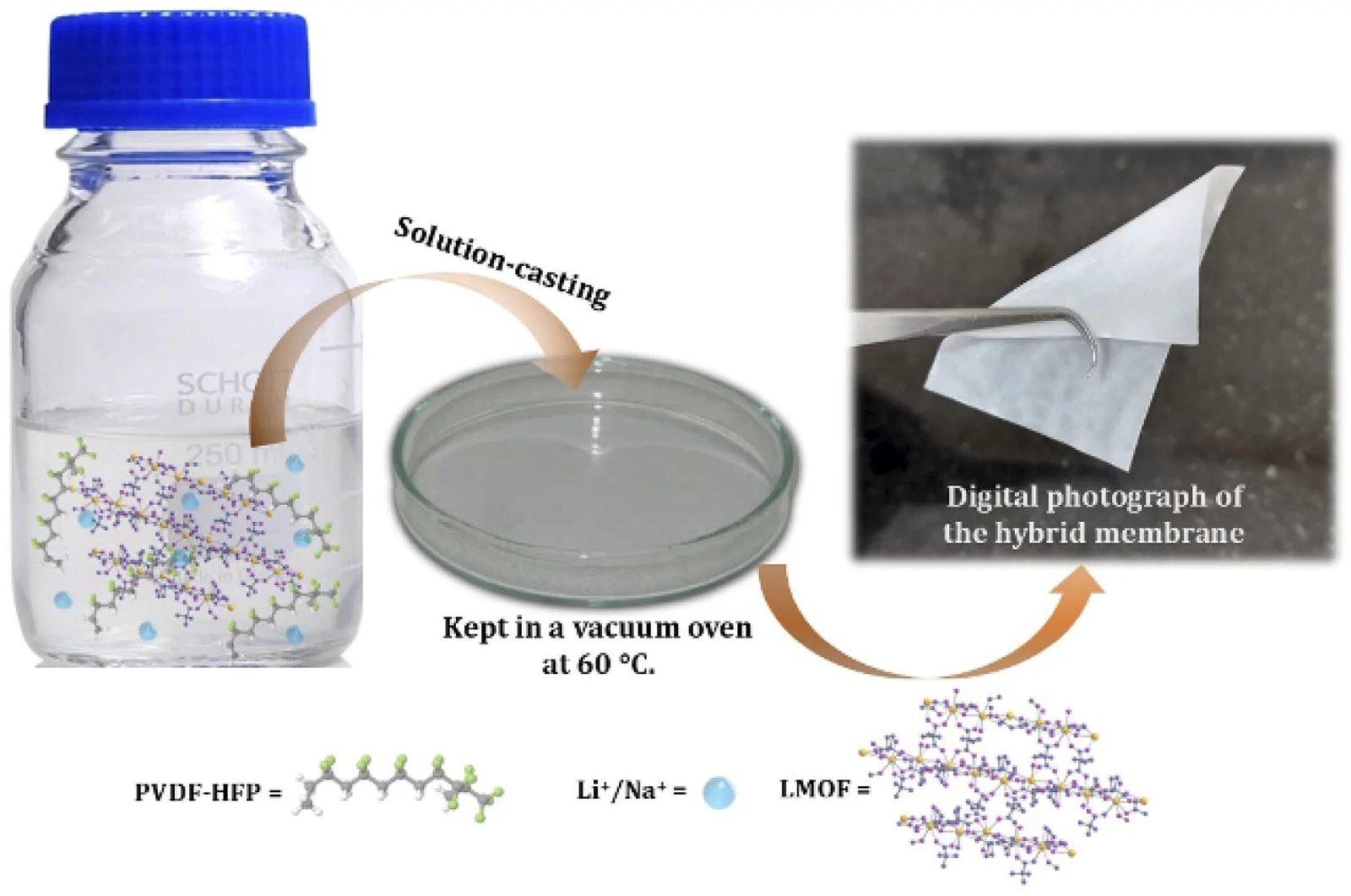
Schematic of the preparation of LMOF-based hybrid membranes.

The crystalline structures of the synthesized LMOFs and their corresponding hybrid membranes were examined using powder X-ray diffraction (PXRD). As shown in Fig. S1, the overall PXRD results confirm the successful integration of the LMOF structure into the PVDF-HFP polymer matrix. The overall FTIR results also confirm the successful formation of the hybrid membranes (Fig. S2). The characteristic absorption bands of PVDF-HFP and metal salts, as well as the stretching vibrations of coordinated carboxylate groups (–COO^−^) of CBDCA, are clearly visible in the FTIR spectra of the hybrid membranes (details in SI). Thermogravimetric analysis (TGA) confirmed that all hybrid membranes exhibit good thermal stability up to approximately 300 °C (Fig. S3). A gradual weight loss of ∼17% up to ∼314 °C is observed for all samples, primarily due to the removal of absorbed and coordinated solvent molecules present within the MOF structure. As the present LMOFs are isostructural, detailed surface characterizations of Dy-MOF@NaHM and MOF@LiHM have been executed *via* Field Emission Scanning Electron Microscopy (FESEM). The surface of Dy-MOF@NaHM exhibits closely packed granular features, which may be due to the dispersion of the NaClO_4_ and Dy-MOF particles within the polymer matrix. The higher magnification image (Fig. 2b) further reveals the presence of an irregular plate-like morphology. Such a morphology suggests the localized clustering of Dy-MOF particles and interactions between the polymer chains and the sodium salt. In contrast, Dy-MOF@LiHM exhibits a more uniform morphology with numerous micro-scale pores distributed across the membrane (Fig. 2c). During the membrane-formation process, the uniform dispersion of Dy-MOF particles and lithium salt within the polymer matrix may contribute to the formation of interconnected pore structures. The higher magnification image (Fig. 2d) clearly illustrates the presence of well-defined pores and channels within the membrane. Such a porous architecture can facilitate easier migration of lithium ions through the membrane matrix.

**Fig. 2:**
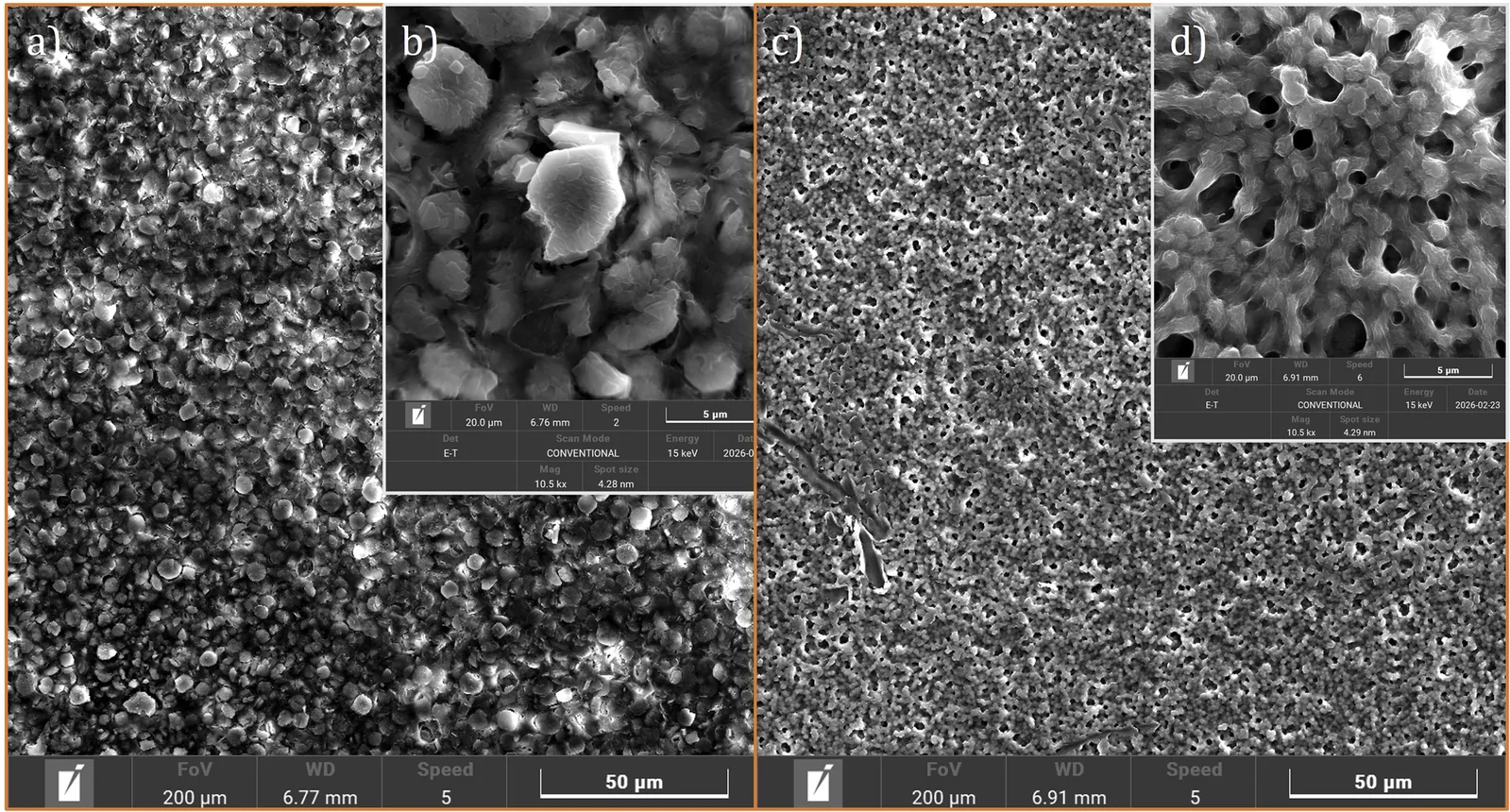
FE-SEM images of (a & b) Dy-MOF@NaHM and (c & d) Dy-MOF@LiHM.

Also, the Dy-MOF@LiHM showed good flame resistance (Table S1). The hybrid membrane did not sustain combustion, and any flame present was rapidly extinguished, demonstrating its self-extinguishing behaviour (Fig. S4).

From the stress–strain profiles (Fig. S5), it is evident that the developed hybrid membranes demonstrate good tensile strength, along with flexible deformation behaviour.

The ionic conductivities of the prepared membranes were investigated using electrochemical impedance spectroscopy. Fig. 3 represents the AC impedance spectra of the (a) Dy/Gd/Tb-MOF@LiHM membranes and (b) Dy/Gd/Tb-MOF@NaHM membranes, where the inset shows the magnified low-frequency region. The Dy-MOF-based hybrid membranes exhibited excellent ionic conductivity for both Li^+^ and Na^+^ ions, reaching the values of 2.537 × 10^−4^ S cm^−1^ and 3.45 × 10^−4^ S cm^−1^, respectively. The conductivity values for the Gd and Tb MOF-based hybrid membranes are a bit lower. However, the overall conductivity of the hybrid membranes lies in the higher range compared with previously reported MOF-based electrolytes (Table S2).^25,27^ Although the lanthanide contraction can significantly influence the proton (H^+^) conduction behaviour of lanthanide-based MOFs.^28–30^ In this work, the conductivity trends do not follow a straightforward relation with the lanthanide ionic radius. So, this variation in conductivity cannot be attributed to the gradual decrease in ionic radii across the lanthanide series.

**Fig. 3:**
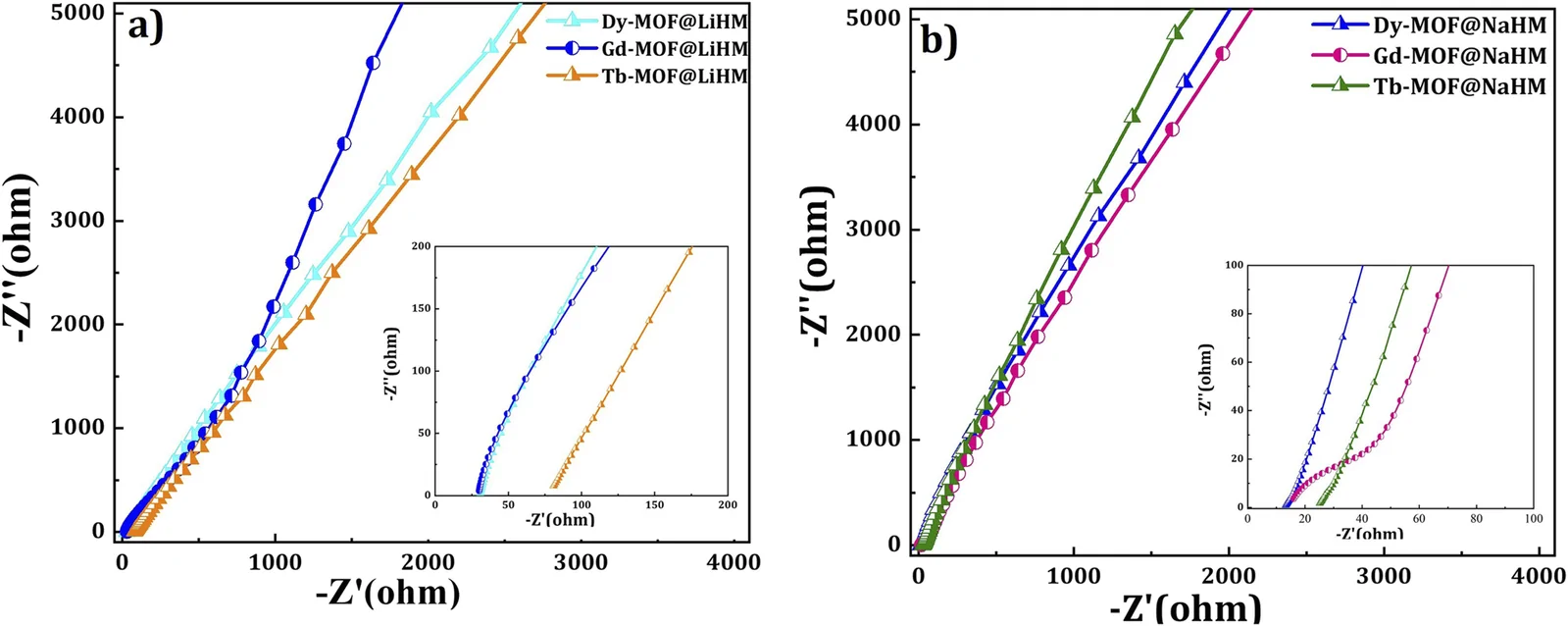
AC impedance spectra of (a) Dy/Gd/Tb MOF@LiHM and (b) Dy/Gd/Tb MOF@NaHM at 25 °C.

In this work, the linker, cyclobutane-1,1-dicarboxylic acid, restricts the framework dimensionality. As a result, the ion can migrate through the interlayer space. A remarkable difference was also observed between the Li^+^- and Na^+^-ion conducting membranes. In all cases, the Na^+^-containing hybrid membranes exhibited higher ionic conductivity than their Li^+^ counterparts. It can be anticipated that lithium ions interact more strongly with polymer chains, functional groups, and coordinated water molecules due to their smaller size and higher charge density. These stronger interactions can limit the mobility of Li^+^ ions within the membrane matrix.^31,32^ In contrast, Na^+^ ions have a relatively larger ionic radius and lower charge density, leading to weaker interactions with the host framework and polymer chains. As a result, Na^+^ ions can migrate more freely through the available ion-conduction pathways in the hybrid membrane, thereby enhancing the overall ionic conductivity. Similar trends in ion mobility have been reported in polymer and solid-state electrolyte systems, where ion-polymer interactions play an important role in determining ionic-transport behaviour.^33,34^

The conductivity of all the hybrid membranes increased with increasing temperature. For instance, the conductivity of Dy-MOF@LiHM increased from 2.537 × 10^−4^ S cm^−1^ at 25 °C to 3.627 × 10^−4^ S cm^−1^ at 55 °C. Similar trends were observed for all other Li- and Na-based hybrid membranes (Table 1 and Fig. S6).

**Table 1:** Ionic conductivity of the hybrid membranes at different temperatures with their activation energies

Hybrid membrane	Conductivity (S cm^−1^) (temperature (°C))	Conductivity (S cm^−1^) (temperature (°C))	Activation energy (eV)
Dy-MOF@LiHM	2.537 × 10^−4^ (25)	3.627 × 10^−4^ (55)	0.097
Gd-MOF@LiHM	1.878 × 10^−4^ (25)	3.584 × 10^−4^ (55)	0.180
Tb-MOF@LiHM	7.992 × 10^−5^ (25)	1.350 × 10^−4^ (55)	0.177
Dy-MOF@NaHM	3.45 × 10^−4^ (25)	6.124 × 10^−4^ (55)	0.160
Gd-MOF@NaHM	2.85 × 10^−4^ (25)	5.303 × 10^−4^ (55)	0.164
Tb-MOF@NaHM	2.66 × 10^−4^ (25)	3.602 × 10^−4^ (50)	0.115

This temperature-dependent enhancement in conductivity indicates improved ion mobility, resulting from increased polymer-chain flexibility and segmental motion at elevated temperatures. The calculated activation energy (*E*_a_) values for all the hybrid membranes lie in the range of ∼0.097 to 0.180 eV, which are relatively low (Table 1 and Fig. S7). Such low activation energies suggest that ion transport within these membranes requires minimal energy and occurs efficiently. The relatively low activation energies (<0.4 eV) obtained from the Arrhenius plots indicate thermally activated ion transport and are consistent with hopping-type ion conduction. However, activation-energy analysis cannot unambiguously establish the ion-transport mechanism for hybrid membranes. Therefore, the ion-hopping proposed here should be regarded only as a plausible interpretation. For the hopping mechanism, ions migrate by jumping between coordination sites within the polymer and MOF framework.^35,36^ This overall ion-transport behaviour makes these hybrid membranes promising candidates for future electrochemical applications.

In summary, we have developed a series of flexible hybrid solid-state electrolyte membranes by integrating 2D lanthanide MOFs with PVDF-HFP, demonstrating a rare combination of high thermal stability, mechanical robustness, and intrinsic self-extinguishing behaviour. Notably, these membranes exhibit excellent dual-ion transport for both Li^+^ and Na^+^ ions. To the best of our knowledge, this is the first demonstration of lanthanide MOF-based hybrid membranes capable of supporting both lithium- and sodium-ion conduction within a unified material platform. So, this work provides a new direction for exploring versatile next-generation solid-state electrolyte materials. Furthermore, the present findings emphasize the urgent need to further explore and develop lanthanide MOF-based hybrid membranes for practical applications in metal-ion batteries.

## Author contributions

Aparna Potdar: material preparation, material characterization, data analysis, investigation, primary draft preparation, *etc.* Vaibhav Phopse: material preparation, material characterization, *etc.* Dev Kumar Thapa: material characterization, investigation, draft preparation, *etc.* Chetana Patil: material characterization, *etc.* Soumava Biswas: conceptualization, data analysis, investigation, final draft preparation, overall supervision, *etc.*

## Conflicts of interest

There are no conflicts to declare.

## Supplementary Material

RA-OLF-D6RA06654B-s001

## Data Availability

The data related to this manuscript have been included in the supplementary information (SI). Supplementary information: Fig. S1–S7 and other experimental details. See DOI: https://doi.org/10.1039/d6ra06654b.
