# Supplementary material for: Observation of high ionic (Li+ and Na+) conductivity in novel hybrid membranes based on 2D lanthanide MOFs
Source: RSC Adv. 2026 Sep 11;16(44):54738–41. doi: 10.1039/d6ra06654b (PMC13568729; doi:10.1039/d6ra06654b)
Supplement: Supplementary file 1 [file RA-016-D6RA06654B-s001.pdf]

Supplementary Information (SI) for RSC Advances.
This journal is © The Royal Society of Chemistry 2026

Supporting Information

Observation of high ionic (Li^+ / Na^+) conductivity in novel hybrid membranes based on 2D lanthanide MOFs

Aparna Potdar, Vaibhav Phopse, Dev Kumar Thapa, Chetana Patil and Soumava Biswas*

Materials and methods:

All chemicals and solvents used in this study were purchased from commercial suppliers and utilized without further purification. Cyclobutane-1,1-dicarboxylic acid, $Gd(NO_3)_3\cdot 6H_2O$, $Dy(NO_3)_3\cdot 6H_2O$, and $Tb(NO_3)_3\cdot 6H_2O$ were obtained from Sigma-Aldrich and used as received. Poly(vinylidene fluoride-co-hexafluoropropylene) (PVDF-HFP) was also procured from Sigma-Aldrich Chemicals. Lithium bis(trifluoromethanesulfonyl)imide (LiTFSI) salt and Sodium perchlorate ($NaClO_4$) was purchased from TCI Chemicals and used without any additional purification.

Experimental Section

Synthesis of $\{[Gd_2(L)_3(H_2O)_2]_n\cdot 2nCH_3OH\cdot 2nH_2O\}$(Gd-MOF)

All the lanthanide-based metal–organic frameworks (MOFs) were prepared by the co-precipitation method, according to the procedure described in earlier reports.[1]The disodium salt of cyclobutane-1,1-dicarboxylic acid (0.752 g, 4 mmol) was dissolved in distilled water (20 mL). In a separate vessel, $Gd(NO_3)_3\cdot 6H_2O$ (1.805 g, 4 mmol) was dissolved in methanol (20 mL). The methanolic solution of $Gd(NO_3)_3\cdot 6H_2O$ was added dropwise to the aqueous ligand solution under continuous stirring at room temperature. The resulting mixture was stirred for 30 min, during which a solid precipitate formed. The product was isolated by filtration, washed thoroughly with distilled water, and dried at room temperature to get Gd-MOF.

Synthesis of $\{[Tb_2(L)_3(H_2O)_2]_n\cdot 2nCH_3OH\cdot 2nH_2O\}$ (Tb-MOF)

Tb-MOF was prepared following the same procedure as described for Gd-MOF, using $Tb(NO_3)_3\cdot 6H_2O$ (1.74 g, 4 mmol) instead of Gd $(NO_3)_3\cdot 6H_2O$. All other reaction conditions and work-up procedures were identical.

Synthesis of $\{[Dy_2(L)_3(H_2O)_2]_n\cdot 2nCH_3OH\cdot 2nH_2O\}$ (Dy-MOF)

Dy MOF was synthesized using the same method as for Gd-MOF, replacing Gd $(NO_3)_3\cdot 6H_2O$ with $Dy(NO_3)_3\cdot 6H_2O$ (1.826 g, 4 mmol). The solvent volumes, reaction time, and isolation procedure remained unchanged.

Characterization Techniques

Different characterization techniques were explored for the structural and morphological elucidation of the materials. The morphological study of MOF@LiHM/NaHM was carried out using a Field Emission Scanning Electron Microscope (FE-SEM, TESCAN, CLARA). The thermal stability study of all hybrid membranes was performed over a wide range of temperatures of 50 –700 °C using TG-DTA (Setsys instrument from Setaram) and N_2 flux (with a heating rate of around 10 ºC min-1). Before measurement, the respective sample was degassed at 80°C for 12 h. Fourier-transform infrared (FT-IR) spectra were measured using a Perkin-Elmer FTIR spectrometer in transmittance mode (within 400 and 4000 cm-1). On a Universal testing system (UTS), the stress-strain testing of the hybrid membranes was conducted to investigate the mechanical strength (STS 248, India). Dumbbell-shaped samples were evaluated for tensile stress and strain in tests at room temperature.

Electrochemical characterization

The ionic conductivity of the prepared hybrid membranes was evaluated using the AC impedance technique. For this purpose, a symmetric SS | GPE | SS (stainless steel | Hybrid membrane | stainless steel) cell configuration was assembled, and electrochemical impedance spectroscopy (EIS) measurements were carried out over a frequency range of 1 MHz to 1 Hz with an applied AC amplitude of 10 mV. The ionic conductivity (σ) of the membranes was calculated using the following relation:

$$\sigma = l/RA$$

where l represents the thickness of the membrane (cm), R corresponds to the bulk resistance obtained from the intercept on the real axis (Z′) of the impedance plot, and A denotes the

effective contact area between the electrode and the electrolyte. The measurements were conducted over a temperature range of 25 °C to 55 °C in order to study the temperature-dependent ionic transport behaviour of the hybrid membranes.

Powder X-ray diffraction (PXRD) measurement

The synthesized lanthanide MOFs and their derived hybrid membranes were characterized by powder X-ray diffraction (PXRD) to evaluate structural integrity and phase purity. As shown in Fig. S1, the experimental diffraction profiles of the synthesized MOFs exhibit peak positions in good agreement with the theoretical patterns simulated from the corresponding CIF data, thereby confirming the successful crystallization and phase-pure formation of the lanthanide-based frameworks.[2] Notably, while the dominant diffraction peaks show strong correspondence, the relatively low intensities of certain minor reflections in the synthesized samples compared to the CIF-simulated patterns can be attributed to finite crystallite size effects, little loss of crystallinity in powder form, slight lattice distortions introduced during solvothermal synthesis, and possible preferred orientation of crystallites during sample preparation.[3]

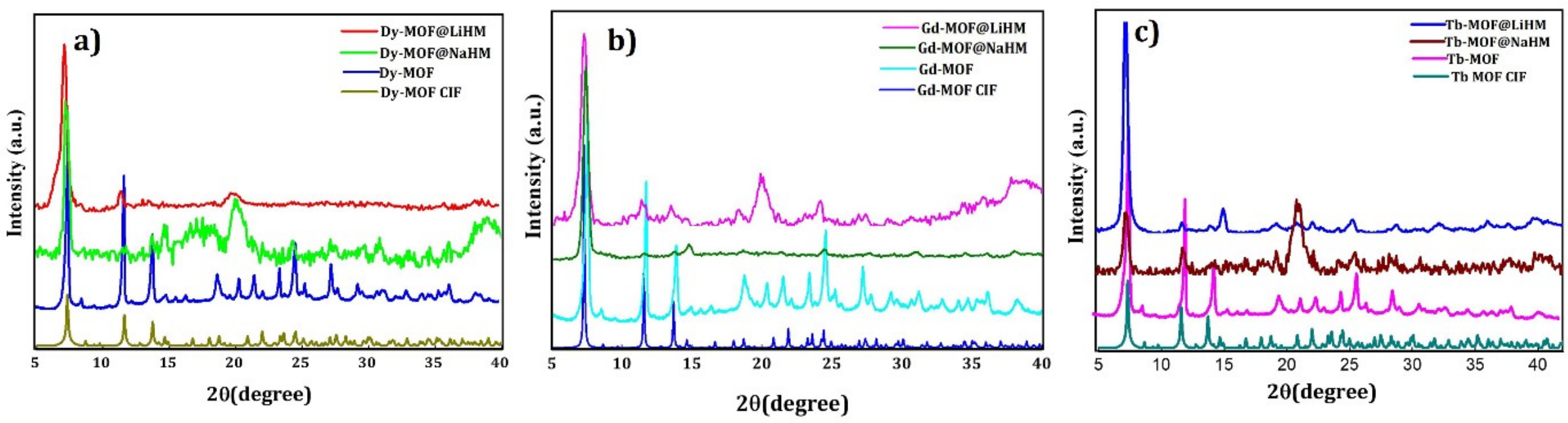

Fig. S1 XRD patterns of a) Dy-MOF CIF(CCDC 993153), Synthesized Dy-MOF, Dy-MOF@LiHM and Dy-MOF@NaHM b) Gd-MOF CIF(CCDC 993152), Synthesized Gd-MOF, Gd-MOF@LiHM and Gd-MOF@NaHM c) Tb-MOF CIF(CCDC1041499), Synthesized Tb-MOF, Tb-MOF@LiHM and Tb-MOF@NaHM.

Incorporation of the MOF particles within the PVDF-HFP polymer matrix in the presence of LiTFSI or $NaClO_4$ salts the resulting hybrid membrane patterns displaying a discernible reduction in overall peak intensities accompanied by moderate peak broadening. Despite this, the characteristic peaks (at low angles) associated with the MOF phases remain identifiable in the membrane patterns, demonstrating that the crystalline framework architecture is retained after polymer encapsulation. The diffuse scattering contributions and broadened baseline features observed in the membrane patterns are ascribed to the semi-crystalline character of the

PVDF-HFP matrix and its interactions with the dissolved ionic salts and dispersed MOF crystalline particles.

Fourier transform infrared (FTIR) spectroscopic study

Fourier Transform Infrared (FTIR) spectroscopy was employed to investigate the chemical structure and interactions within the prepared hybrid membranes (Fig. S2). Fig. S2 (a) represents Dy-, Gd-, and Tb-MOF-incorporated Li-based membranes (LiHM), while Fig. S2 (b) corresponds to the respective Na-based membranes (NaHM). All membranes consist of PVDF-HFP as the polymer matrix, LiTFSI or $NaClO_4$ as the salt, and Dy/Gd/Tb-CBDCA MOF as the filler. Peaks in the region of ~1400–1200 cm^{-1} are assigned to $–CF_2$ stretching vibrations, while bands around ~830–880 cm^{-1} correspond to the β-phase of PVDF-HFP, confirming the retention of its electroactive structure after membrane formation.[4-6] The incorporation of ionic salts is confirmed by bands in the range of ~1000–1100 cm^{-1}, attributed to S=O and S–N–S vibrations of LiTFSI. [7]In the case of $NaClO_4$, characteristic peaks observed at ~1081 cm^{-1} and ~623 cm^{-1} correspond to the asymmetric stretching and bending vibrations of the ClO_4^- anion.[8] The slight shifts in these peaks indicate interactions between the salt ions and the polymer matrix. The presence of Dy-, Gd-, and Tb-based MOFs is evidenced by bands in the region of ~600–800 cm^{-1}, which are assigned to metal–oxygen (Dy–O, Gd–O, Tb–O) vibrations.[9, 10] Additionally, bands observed in the range of ~1543–1630 cm^{-1} are attributed to the stretching vibrations of coordinated carboxylate groups ($–COO^-$) from the CBDCA linker, confirming the incorporation of the MOF structure within the polymer matrix. A broad absorption band around ~3554 cm^{-1} is associated with O–H stretching vibrations, arising from adsorbed moisture and hydrogen-bonded hydroxyl groups within the MOF and polymer network. No new peaks or significant disappearance of characteristic bands are observed, indicating that the membrane fabrication process preserves the chemical structure of all components. However, slight variations in peak position and intensity suggest intermolecular interactions and coordination among PVDF-HFP, salts, and MOF fillers. Furthermore, minor differences between Li-based and Na-based membranes are observed, which can be attributed to variations in ionic size and interaction strength of Li^+ and Na^+ ions with the polymer chains and MOF framework. Overall, the FTIR results confirm the successful formation of a compatible hybrid system with strong interactions among the components, which is expected to favour improved ion transport properties.

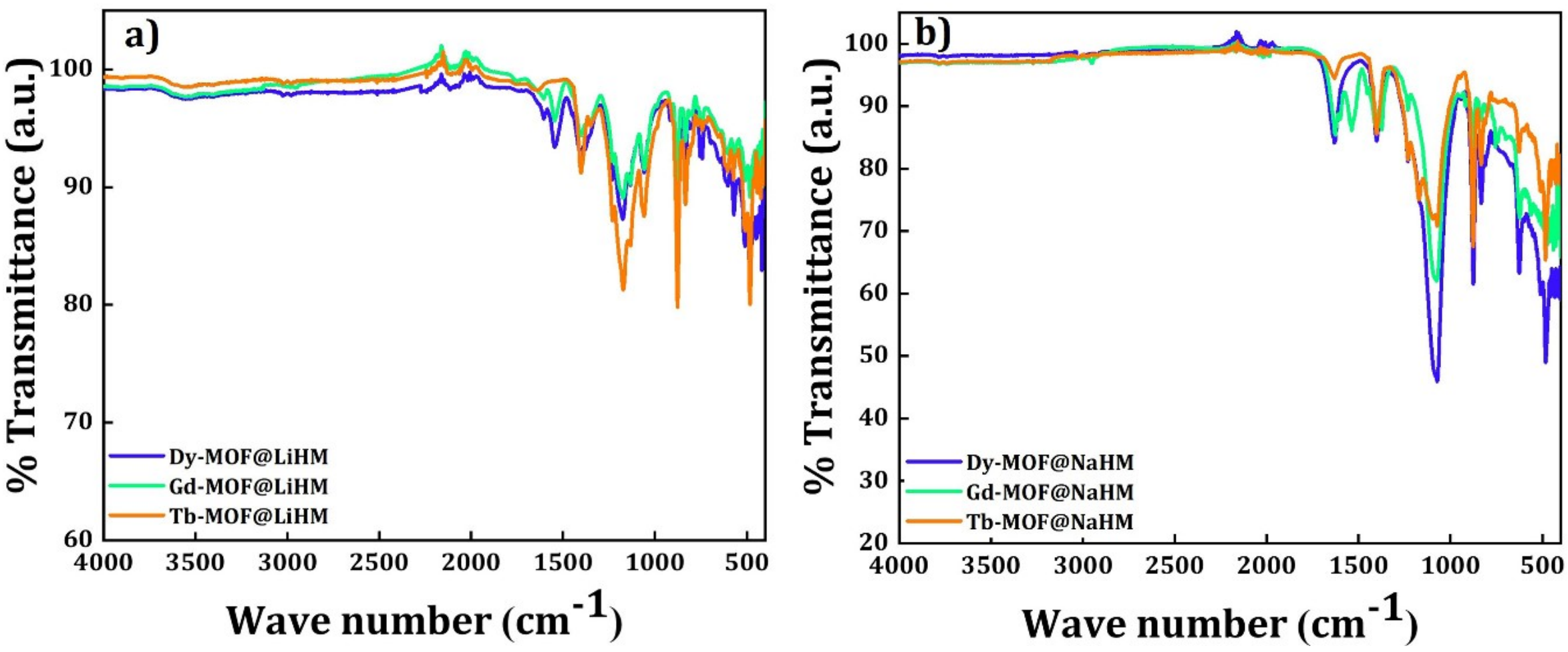

Fig. S2 FTIR spectra of Dy-, Gd-, and Tb-based MOF hybrid membranes incorporated with lithium and sodium salts.

Thermogravimetric analysis

The thermal stability of all the hybrid membranes was analysed using TGA (Fig. S3). A small and gradual weight loss of around 17% up to 314 °C is observed for all samples, which can be attributed to the removal of absorbed and coordinated water molecules present within the MOF structure. After 508 °C, all membranes show a major weight loss of approximately 58%, corresponding to the decomposition of the polymer matrix and Lithium or sodium salt. On the other hand, the Gd-MOF@LiHM and Tb-MOF@LiHM membranes exhibit a stepwise degradation pattern. An intermediate weight loss of about 32% occurs near 341 °C, followed by a further weight loss of around 48% at approximately 438 °C, which can be associated with the progressive decomposition of the polymer chains and MOF structure. Overall, the hybrid membrane demonstrates adequate thermal stability, indicating its suitability for battery application.

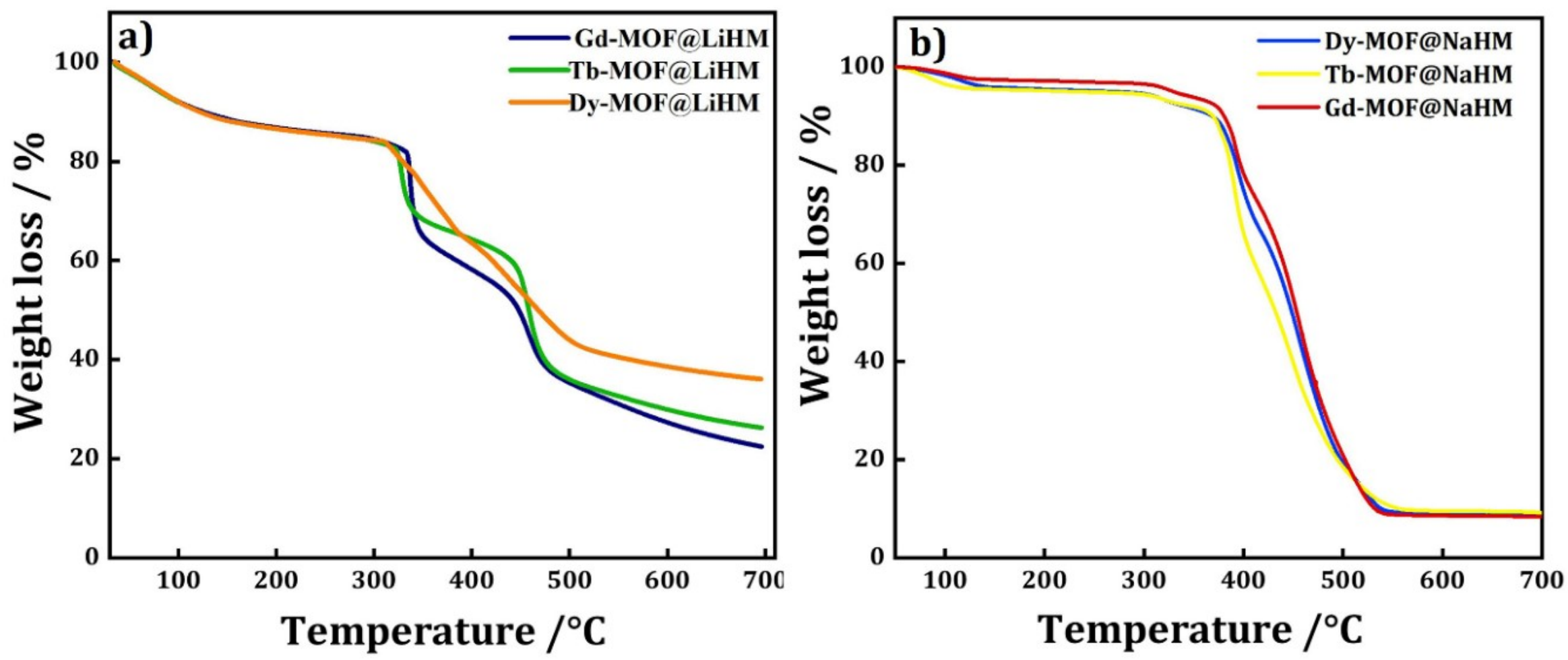

Fig. S3 a) TGA curves of Dy/Gd/Tb MOF based Li hybrid membrane, b) TGA curves of Dy/Gd/Tb MOF based Na hybrid membrane.

Flame test

The fire safety of the prepared membranes was evaluated by conducting a vertical flammability test following the IS 15061:2002 standard, using 5 × 1 cm strips of each sample (Fig. S4). When exposed to a direct flame, the commercial Celgard (PP) separator caught fire within approximately 1–2 seconds and was completely consumed in nearly 4–5 seconds[11, 12], leaving behind absolutely no residual material, a 0% mass retention which reflects the inherently poor thermal stability of polyolefin-based separators. In stark contrast, the Dy-MOF@LiHM hybrid membrane did not show any sustained ignition upon flame contact. Once the flame source was withdrawn, the membrane self-extinguished within just 2 seconds[13], yielding a normalized self-extinguishing time (SET) of 44.4 s g^{-1} and retaining as much as 93% of its original mass after the test.[11, 14] The membrane also preserved its structural integrity and shape completely, further confirming the robust flame-retardant character imparted by the Dy-MOF framework and presence of C-F bond of polymer. These findings, as summarized in Table S1 and illustrated in Fig. S4 (c and d), clearly demonstrate that the Dy-MOF@LiHM membrane offers a substantially improved level of fire safety compared to the conventional Celgard separator, making it a more reliable and thermally secure candidate for deployment in lithium-ion and sodium-ion battery systems.

TableS1: Vertical flammability test results (IS 15061:2002)

**Parameter**	**Celgard (PP)**	**Dy-MOF@LiHM**
Test standard	IS 15061:2002	IS 15061:2002
Sample dimensions (cm)	5 × 1	5 × 1
Sample mass (g)	0.0074	0.045
Time to ignition (s)	~1–2	No sustained ignition
Total burn time (s)	~4–5	—
SET after flame removal (s)	Complete combustion	2
SET (s g^{-1})	0	44.4
Residual mass (%)	0	93
Flame behaviour	Propagates rapidly	Self-extinguishing
Shape retained after test	No	Yes

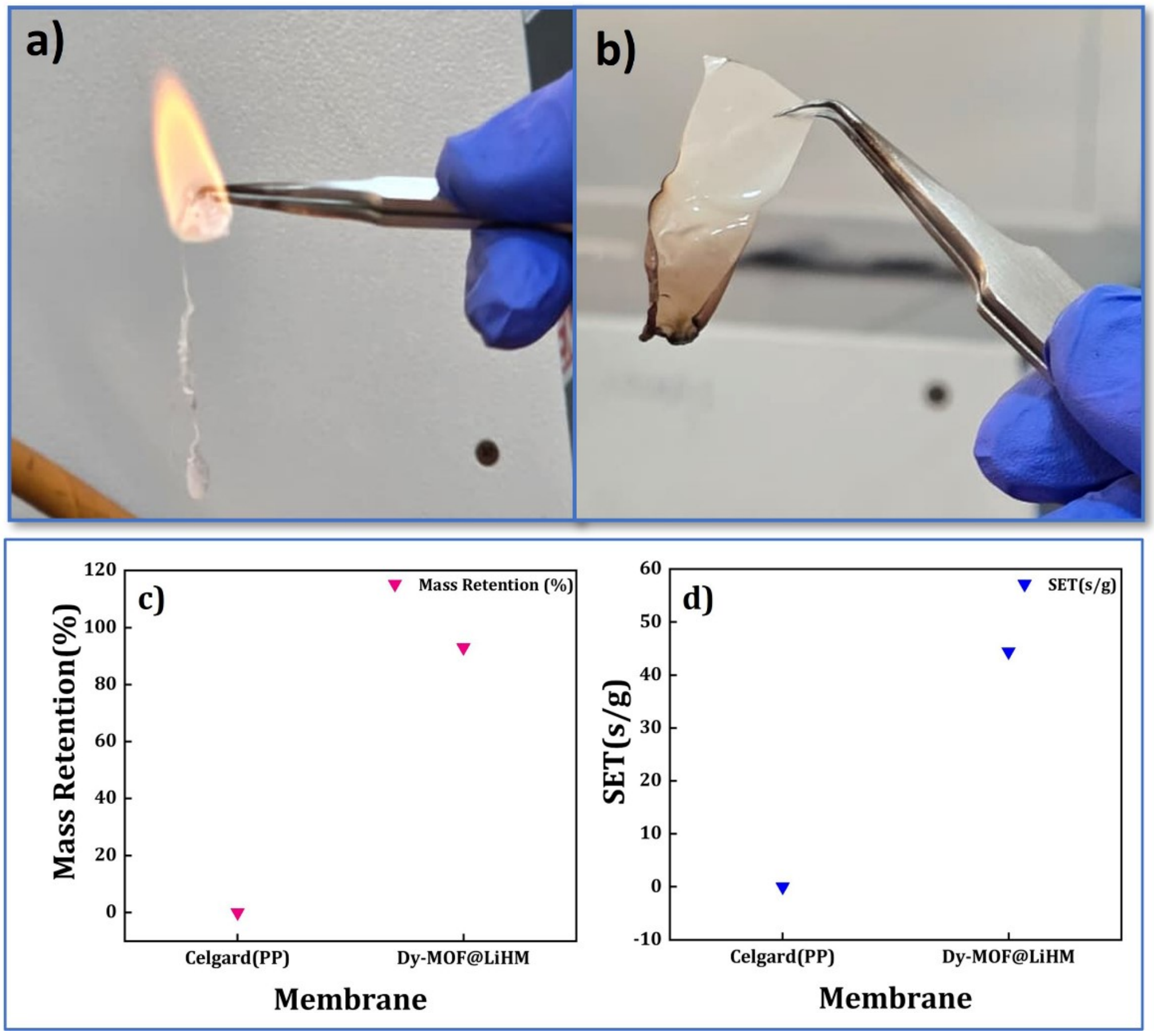

Fig. S4 Digital photograph of flame-retardant test for a) Celgard membrane and b) Dy-MOF@LiHM membrane, c) Plot of Mass retention (%) Vs Membrane, d) Plot of SET(s/g) Vs Membrane

Mechanical property study

The stress–strain behaviour of the developed hybrid membranes is presented in Fig.S5. The curves in Fig.S5 (a) correspond to the Li^{+}-based membranes, while those in Fig.S5 (b) represent the Na^{+}-based membranes. For the Li-containing membranes, the curves show an initial sharp rise in stress followed by a gradual and stable deformation region. These membranes can withstand large strain values (up to ~500%), indicating good flexibility and ductile nature. Among the different compositions, the Dy-based membrane shows slightly improved strength, which may be due to better interaction between the polymer chains and the MOF particles. In the case of the Na-containing membranes, a similar trend is observed with a rapid increase in stress at low strain, reaching relatively high strength values (~15–17 MPa). Although these membranes show lower elongation compared to the Li-based systems, they still maintain stable mechanical behaviour without sudden failure. For context, the commercial Celgard 2400 (PP) separator shows a tensile strength of approximately 139 MPa in the machine direction (≈13.7 MPa in the transverse direction) with limited flexibility under transverse loading,[15-17] while

MOF-based composite membranes reported in literature achieve tensile strengths in the range of 6.63–21.6 MPa with elongation values spanning 232–500% depending on the polymer matrix.[3, 18] The mechanical response of our Dy-MOF@LiHM membrane falls well within this range, confirming that the MOF incorporation provides adequate reinforcement within the polymer matrix without compromising flexibility. This suggests that the incorporation of MOF fillers provides sufficient reinforcement within the polymer matrix. These results confirm that the membranes possess adequate mechanical integrity and are suitable for use as hybrid membranes in battery applications.

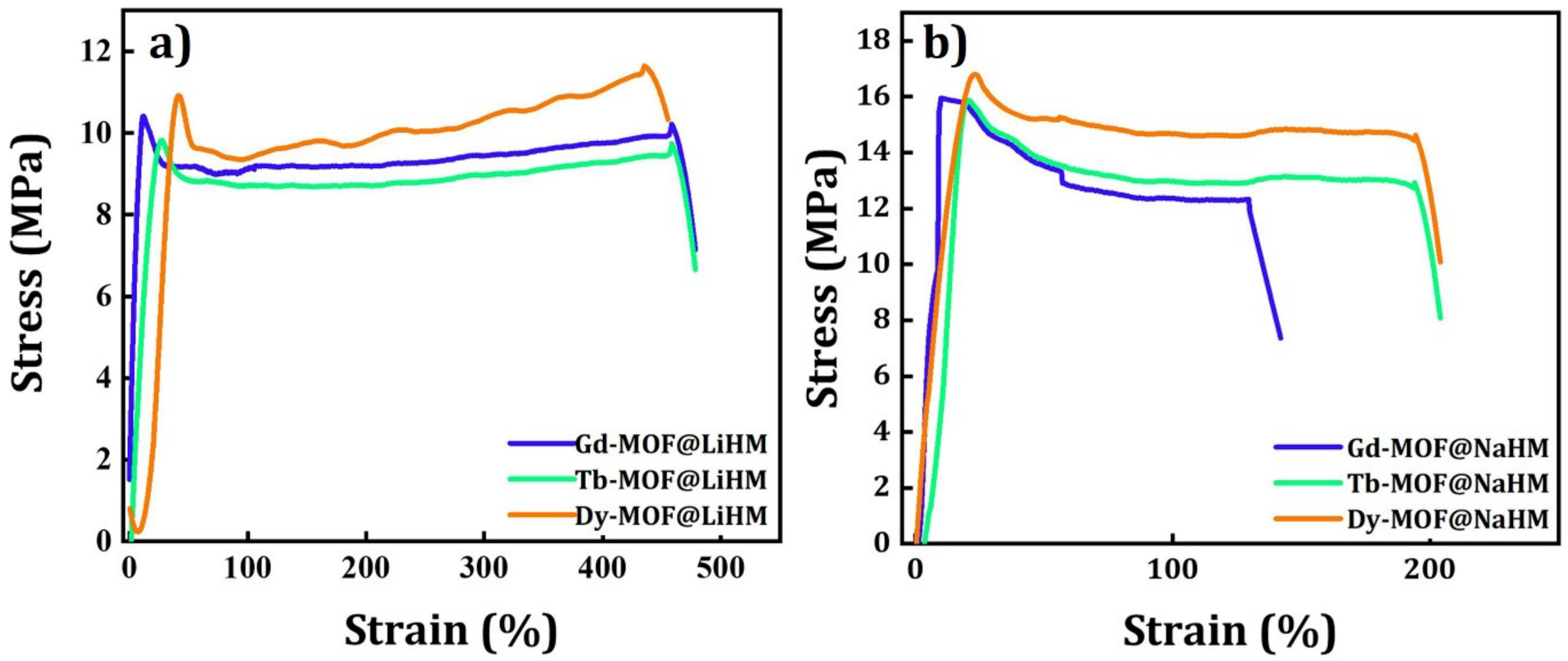

Fig. S5 Stress–strain profiles illustrating the mechanical performance of Li^+ and Na^+ ion incorporated hybrid membranes.

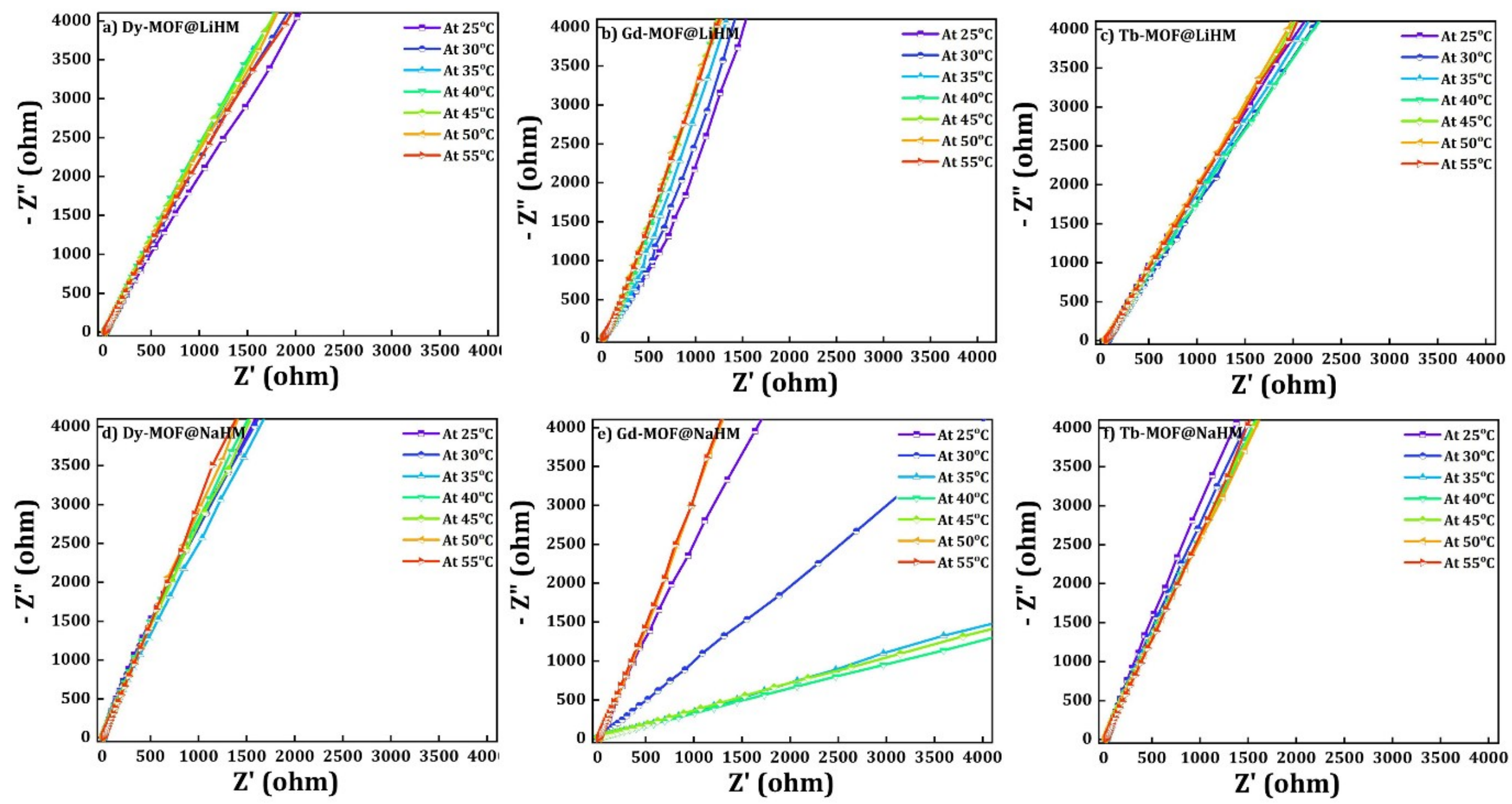

Fig. S6 Nyquist plots of hybrid membrane at a different temperature

Estimation of activation energy (E_a)

The variation of ionic conductivity with temperature was analysed using Arrhenius plots (Fig. S6), where log(σ) is plotted against 1000/T. The obtained linear relationship indicates that the conductivity follows Arrhenius-type behaviour, which can be expressed as:

$$\sigma \quad = \sigma 0 exp\,(- Ea \,/\, kT\,)$$

where σ_0 is the pre-exponential factor, E_a represents the activation energy for ion transport, k is the Boltzmann constant, and T is the absolute temperature in Kelvin.

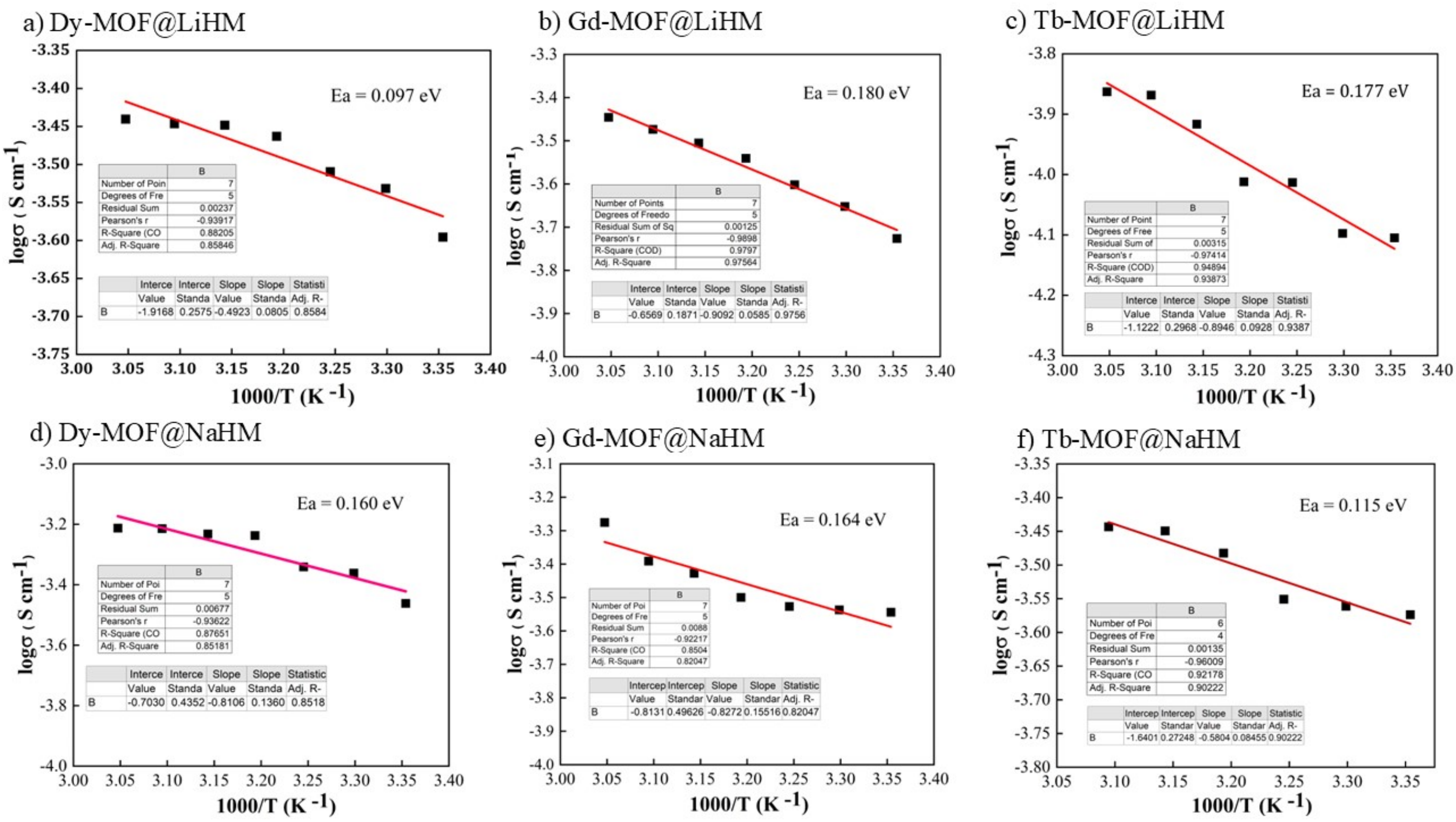

Fig. S7 Arrhenius plot of the ionic conductivity of hybrid membrane of the cell SS| Dy/Gd/Tb MOF @ Li/Na HM|SS.

Table S2. The comparative literature data on the selected reported system

MOF	Polymer	σ (Scm^{-1})/T (°C)	Ref
Li-IL@HKUST-1	PEO	1.20×10^{-4} / 30	[19]
$Ni_3(BTC)_2$	PVDF-HFP	1.4×10^{-4}/30	[20]
$UiO\text{-}66\text{-}NH_2@SiO_2$	PEO	2.89×10^{-5}/60	[7]
ZIF-67	PEO	1.4×10^{-6}/25	[21]
Ni (BDC)	PVDF-HFP	1.5×10^{-4}/25	[22]
ZIF-8	PVDF-HFP	2.8 X 10^{-4}/30	[23]
**Dy-MOF@LiHM**	PVDF-HFP	2.537 X 10^{-4}/25<br>3.627 X 10^{-4}/55	**This work**
**DyMOF@NaHM**	PVDF-HFP	3.45 X 10^{-4}/25<br>6.124 X 10^{-4}/55	

References

1. S. Biswas, H. S. Jena, S. Sanda and S. Konar, *Chemistry – A European Journal*, 2015, **21**, 13793-13801.
2. C. F. Holder and R. E. Schaak, *ACS Nano*, 2019, **13**, 7359-7365.
3. P. Cheng, S. Liu, X. Jia, Y. Jiang and X. Zhang, *Nano Letters*, 2025, **25**, 6152-6159.
4. A. S. Potdar, R. B. Kale, R. S. Ballal, J. Ambekar, R. S. Kalubarme, N. D. Khupse, B. B. Kale and M. V. Kulkarni, *New Journal of Chemistry*, 2025, **49**, 3227-3235.
5. L. N. Sim, S. R. Majid and A. K. Arof, *Vibrational Spectroscopy*, 2012, **58**, 57-66.
6. A. Potdar, S. Biswas, D. K. Thapa, B. Kale, M. V. Kulkarni and M. Chavali, *European Journal of Inorganic Chemistry*, 2025, **28**, e202500075.
7. N. Angulakshmi, Y. Zhou, S. Suriyakumar, R. B. Dhanalakshmi, M. Satishrajan, S. Alwarappan, M. H. Alkordi and A. M. Stephan, *ACS omega*, 2020, **5**, 7885-7894.
8. M. R. Mettu, A. Mallikarjun, M. J. Reddy and J. S. Kumar, *Rasayan J. Chem.*, 2022, **15**, 2188-2196.
9. H. Brunckova, E. Mudra, L. Rocha, E. Nassar, W. Nascimento, H. Kolev, A. Kovalcikova, Z. Molcanova, M. Podobova and L. Medvecky, *Applied Surface Science*, 2021, **542**, 148731.
10. B. Zhang, P. Huang, J. Chen, X. Dang, Y. Hu, Y. Ai, D. Zheng and H. Chen, *Applied Surface Science*, 2020, **504**, 144504.
11. Y. Guo, D. Zhou, R. Li, D. Li, W. Zhao, L. Pang, Z. Shi, W. Liu, J. Su, T. Zhou and G. Chen, *Journal of Energy Storage*, 2024, **76**, 109585.
12. M. Das, P. S. Das, N. C. Pramanik, R. N. Basu and M. Wasim Raja, *ACS Omega*, 2023, **8**, 21315-21331.
13. J.-H. Kim, J.-H. Hyun, S. Kim, W. H. Park and S.-H. Yu, *Advanced Energy Materials*, 2025, **15**, 2500587.
14. J. Fu, H. Wang, Z. Du, Y. Liu, Q. Sun and H. Li, *SmartMat*, 2023, **4**, e1182.
15. X. Wang, X. Huang, Z. Ji, W. Hu, H. Sheng and X. Li, *Polymer*, 2023, **283**, 126274.
16. J. Xu, L. Wang, J. Guan and S. Yin, *Materials & Design*, 2016, **95**, 319-328.
17. A. Sheidaei, X. Xiao, X. Huang and J. Hitt, *Journal of Power Sources*, 2011, **196**, 8728-8734.
18. T. Zhao, Y. Bi, J. Chen, J. Yu, S. Peng, F. Luo and Y. Chen, *Safety*, 2025, **11**, 111.
19. Z. Wang, H. Zhou, C. Meng, W. Xiong, Y. Cai, P. Hu, H. Pang and A. Yuan, *ACS Applied Energy Materials*, 2020, **3**, 4265-4274.

20. S. Suriyakumar, M. Kanagaraj, N. Angulakshmi, M. Kathiresan, K. S. Nahm, M. Walkowiak, K. Wasiński, P. Półrolniczak and A. M. Stephan, *RSC advances*, 2016, **6**, 97180-97186.
21. Y. Xu, R. Zhao, J. Fang, Z. Liang, L. Gao, J. Bian, J. Zhu and Y. Zhao, *Frontiers in Chemistry*, 2022, **10**, 1013965.
22. A. Potdar, S. Biswas, D. K. Thapa, B. Kale, M. V. Kulkarni and M. Chavali, *European Journal of Inorganic Chemistry*, 2025, **28**, e202500075.
23. J. Zhang, D. Li, B. Wen, Y. Wang, Z. Lei, C. Yin, L. Fan and G. Yang, *Journal of Materials Science: Materials in Electronics*, 2023, **34**, 938.
